# Calcific Aortic Valve Disease Assessment: Strengths and Challenges

**DOI:** 10.1111/echo.70261

**Published:** 2025-08-09

**Authors:** Diana‐Elena Floria, Mariana Floria, Daniela Maria Tanase

**Affiliations:** ^1^ Department of Internal Medicine Faculty of Medicine University of Medicine and Pharmacy “Grigore T. Popa” Iasi Romania; ^2^ Emergency Clinical Hospital “Saint Spiridon Iasi Romania

1

Calcific aortic valve disease (CAVD) is a progressive remodeling of the aortic valve leaflets which include thickening, fibrosis, and mineralization, evolving from atherosclerosis to aortic sclerosis and calcific aortic stenosis. Age is an important and non‐modifiable risk factor for CAVD, along with dyslipidemia, hypertension, plasma lipoprotein(a) levels, obesity, and diabetes mellitus, which are modifiable factors. The assessing of calcific aortic valve is extremely important, especially, because CAVD is a progressive condition that can lead to aortic stenosis; it has serious clinical consequences if left untreated. The long‐term outcomes of patients with aortic sclerosis and mild aortic stenosis detected at the time of clinically indicated echocardiography are important. Patients with aortic sclerosis are >25 times more likely to require aortic valve intervention than those with no CAVD, and follow‐up echocardiography should be considered within 3–4 years in those with mild aortic stenosis [1]. Appropriate assessment of CAVD is very important equally, for patient diagnostic and follow‐up, but also for treatment and prognosis. The recently published meta‐analysis of Huang et al. [2] concluded that the ultrasound demonstrates high accuracy in diagnosing senile CAVD; however, misdiagnosis or missed diagnosis can happened because echocardiography, even it is the golden standard imaging modality, it does not completely eliminate this possibility. Therefore, even echocardiography is considered the gold standard imaging modality for assessing CAVD it has some strengths and challenges.

Accurate assessment of CAVD is essential for diagnosis, monitoring, and treatment planning. The bi‐ or tridimensional transthoracic and transesophageal echocardiography allows the measurement of all parameters for quantifying the severity CAVD by providing structural (valve morphology) and functional (hemodynamic) information [3]. The following data can be obtained:

1. Structural information about the aortic valve are:
2. Valve morphology: the valve leaflets or cusps can be thickened, calcified, and with reduced mobility.
3. Valve mobility: the operator should determine whether the valve is fixed or restricted due to heavy calcification, and if affects both, opening (stenosis) and closing (regurgitation) aortic valve.
4. Valve calcification: these can appear as bright echogenic areas with acoustic shadowing. The quantification of calcification though echocardiography is limited. The following semi‐quantitative grading can be applied:
5. Mild: Small bright echoes on leaflet tips.
6. Moderate: More extensive echoes with some shadowing.
7. Severe: Dense, extensive calcification with significant acoustic shadowing and immobility. In addition, the evaluation by cardiac computer tomography may be used adjunctively to quantify valve calcium (e.g., Agatston score). Besides the aortic valve, these calcifications can be on mitral (annulus annular or leaflet tips).
8. Hemodynamic information about aortic valve are obtained by:
9. Color Doppler which detects higher velocities due to reduced opening of the aortic valve or the association of regurgitant jets due to valve incorrect from calcification.
10. Continuous‐wave Doppler measures transvalvular pressure gradients.

The appropriate assessing of CAVD is crucial for early diagnosis, risk assessment, monitoring progression, and deciding on life‐saving interventions. The echocardiography is the key tool because it provides: valve area measurement, peak and mean pressure gradients, flow velocity, and structural visualization of extent of calcification. It allows to identify the progression to aortic stenosis; the risk of heart failure or sudden cardiac death (risk stratification based on valve gradients, valve area, and symptoms, patients with severe symptomatic aortic stenosis being at risk for syncope, angina, and sudden death); the monitoring for intervention timing, when to refer for surgery or transcatheter aortic valve implantation by follow‐up intervals (e.g., every 6–12 months for severe aortic stenosis, because in these cases, without intervention, 2‐year mortality can exceed 50% once symptoms develop) [3]. However, in asymptomatic but severe aortic stenosis, even without symptoms, severe aortic stenosis may require intervention if: left ventricular function is impaired, there is very high aortic velocity (>5.0 m/s) or elevated brain natriuretic peptide levels or abnormal stress testing.

A multimodality and multiparametric approach by combining structural findings with Doppler data, and clinical symptoms to grade the severity and guide intervention decisions (e.g., valve replacement) is mandatory in CAVD [1]. In clinical practice there are some tips and tricks which can improve precision and avoid common pitfalls in echocardiographic evaluation of CAVD. First of all, it is recommended to use multiple acoustic windows (apical, right parasternal, suprasternal). Peak aortic jet velocity may be underestimated if the Doppler beam is not aligned with the flow. Non‐apical views (e.g., right parasternal or suprasternal) can be used, if flow is eccentric or gradients seem low.

Secondly, when using the continuity equation for aortic valve area left ventricular outflow tract diameter should be measured in mid‐systole (from inner edge to inner edge), with zoom, in parasternal long axis view for precision. In addition, pulsed‐wave Doppler is used in the left ventricular outflow tract, and continuous‐wave Doppler across the aortic valve. Do not forget that the errors in left ventricular outflow tract diameter square the mistake.

Thirdly, the aortic valve area must be indexed to body surface area in small or large patients. In high‐gradient aortic stenosis, because larger aortic valves are associated with higher Agatston scores, CAVD should be indexed to aortic valve size in addition to sex [4].

Fourthly, some patients have severe aortic stenosis but low gradients due to low‐flow, low‐output (left ventricular ejection fraction less than 50%) and paradoxical low‐flow (left ventricular preserved but small stroke volume). These cases are so called low‐flow, low‐gradient aortic stenosis. In clinical practice, if we suspect such a case, stroke volume index (<35 mL/m^2^ = low flow) calculation is necessary. In addition, we must consider dobutamine stress echo if left ventricular ejection fraction is reduced, to distinguish true severe versus pseudo‐severe aortic stenosis.

Fifthly, estimation of valve calcification is recommended. Severe calcification causes bright, dense echoes with acoustic shadowing, often visible in parasternal long axis. Although echocardiography is semi‐quantitative, cardiac computer tomography can objectively measure valve calcium (Agatston score). A value of 2000 AU (men) or >1200 AU (women) means severe aortic stenosis [5].

Sixthly, the clinician should correlate the echocardiographic parameters with symptoms and left ventricular function. This implies always assessing left ventricular systolic and diastolic function, the degree of hypertrophy, evaluating symptoms (angina, syncope, dyspnea) and brain natriuretic peptide levels, considering stress testing in asymptomatic severe aortic stenosis to unmask symptoms.

Finally, serial follow‐up is recommended: for moderate aortic stenosis an echocardiography every 1–2 years and for severe aortic stenosis, an echocardiography every 6–12 months, or sooner if symptoms change [3]. It is strongly recommended to use the same machine and sonographer, when possible, for consistent comparisons. In conclusion, there are some pitfalls which can be avoided (Table 1).

**TABLE 1:** The pitfalls and possibilities of avoidance in the assessment of calcific aortic valve disease severity.

Pitfall	How can it be avoided
Inaccurate left ventricular outflow tract measurement	Zoomed parasternal long axis view in mid‐systole
Underestimating gradients	Use multiple windows and align Doppler beam
Ignoring calcification extent	Use cardiac computer tomography when echo unclear
Misclassifying low‐flow aortic stenosis	Use stroke volume index, aortic valve area index, and consider dobutamine echo

Tridimensional echocardiography helps clarify diagnosis, severity grading, and treatment planning in CAVD [6]. Comparing with 2D echocardiography, this allows directly measurement by planimetry of the aortic orifice; is better for assessing leaflet restriction, asymmetry; shows more accurate elliptical shape and improves aortic valve area calculation; have better spatial resolution; identifies distribution of calcium and which leaflets are most affected; allows the real‐time, full motion of all leaflets improving the detection of asymmetric restriction. The strengths of bidimensional echocardiography in CAVD are: widely available and quick to perform; is an excellent imaging method for Doppler‐based assessment (maximal velocity, mean gradient), for aortic valve area by continuity equation, regular follow‐up and initial screening. On the contrary, the strengths of tridimensional echocardiography in CAVD are the following [6]: it is better for anatomical definition (valve orifice shape and area, asymmetrical leaflet restriction, leaflet numbering and localization; it is a more accurate imaging method in complex or borderline cases and it is useful in procedural planning. In conclusion, in clinical practice we can use bidimensional echocardiography for standard assessment and Doppler flow quantification, and tridimensional echocardiography when you need detailed anatomic and spatial information, especially in complex or inconclusive cases of CAVD.

In the assessment of CAVD, echocardiography and computed tomography offers complementary strengths [5]. Echocardiography is preferred as first‐line imaging for all suspected cases of aortic stenosis. In addition, it is essential for grading aortic stenosis, being the best for monitoring disease progression over time. However, computer tomography is recommended when echocardiography is inconclusive (because of poor acoustic windows), to confirm the severity of aortic stenosis in low‐flow, low‐gradient cases, for transcatheter aortic valve replacement planning (precise annular sizing, calcium mapping, vascular access) or when quantitative calcium scoring is needed.

The aortic valve calcification score can be measured on the non‐contrast computer tomography; it is defined as all the calcifications filed on the valve leaflet, on the aortic sinus of Valsalva and on the aortic valve annulus. It seems that, the treatment with vitamin K antagonists as anticoagulant is associated with increased aortic valve calcification score, while a similar association is not true for new oral anticoagulants. For every year treated with vitamin K antagonists, the aortic valve calcification score increased, on average, by 6% [7].

A 2020 consensus statement from the British Society of Cardiovascular Imaging/British Society of Cardiac Computed Tomography and British Society of Thoracic Imaging recommends reporting and visually quantifying aortic valve calcification (and coronary artery calcification) on all non‐contrast and contrast‐enhanced, non‐gated chest computer tomographies, irrespective of indication. This recommendation can allow the identification of aortic stenosis in 16% of patients [8]. The most important is the fact that severe aortic valve calcification is an independent predictor of all‐cause mortality.

In the assessment of CAVD, echocardiography and computed tomography are complementary imaging modalities; echocardiography is more useful for hemodynamics and regular follow‐up and computer tomography for precise calcium burden, anatomy, and pre‐procedural planning. In clinical practice is advisable to start with echocardiography which assess gradients, valve area and the flow. If seems to be a low‐gradient or borderline aortic stenosis we can consider cardiac computer tomography. The last, can confirm the severity of CAVD using calcium scoring. For percutaneous valve replacement candidates, contrast‐enhanced computer tomography is mandatory.

## Conflicts of Interest

The authors declare no potential conflict of interest.
